# Comparative Metabolomic Profiling of Horse Gram (*Macrotyloma uniflorum* (Lam.) Verdc.) Genotypes for Horse Gram Yellow Mosaic Virus Resistance

**DOI:** 10.3390/metabo13020165

**Published:** 2023-01-23

**Authors:** Sudhagar Rajaprakasam, Priyanka Shanmugavel, Vanniarajan Chockalingam, Souframanien Jegadeesan, Tnpalayam Krishnaswamy Sukirtha Latha, Saravanan Naaganoor Ananthan, Raveendran Muthurajan, Selvaraju Kanagarajan

**Affiliations:** 1 Agricultural College and Research Institute, Tamil Nadu Agricultural University, Coimbatore 641003, Tamil Nadu, India; 2 Centre for Plant Breeding and Genetics, Tamil Nadu Agricultural University, Coimbatore 641003, Tamil Nadu, India; 3 Anbil Dharmalingam Agricultural College and Research Institute, Tamil Nadu Agricultural University, Trichy 620027, Tamil Nadu, India; 4 Bhabha Atomic Research Centre, Trombay, Mumbai 400085, Maharashtra, India; 5 Centre for Plant Protection Studies, Tamil Nadu Agricultural University, Coimbatore 641003, Tamil Nadu, India; 6 Sugarcane Research Station, Tamil Nadu Agricultural University, Melalathur, Vellore 635806, Tamil Nadu, India; 7 Directorate of Research, Tamil Nadu Agricultural University, Coimbatore 641003, Tamil Nadu, India; 8 Department of Plant Breeding, Swedish University of Agricultural Sciences, P.O. Box 190, 234 22 Lomma, Sweden

**Keywords:** horse gram, metabolomics, resistance, yellow mosaic virus

## Abstract

Horse gram (*Macrotyloma uniflorum* (Lam.) Verdc.) is an under-utilized legume grown in India. It is a good source of protein, carbohydrates, dietary fiber, minerals, and vitamins. We screened 252 horse gram germplasm accessions for horse gram yellow mosaic virus resistance using the percent disease index and scaling techniques. The percentage values of highly resistant, moderately resistant, moderately susceptible, susceptible, and highly susceptible were 0.34, 13.89, 38.89, 46.43, and 0.34, respectively. Repetitive trials confirmed the host-plant resistance levels, and yield loss was assessed. The present disease index ranged from 1.2 to 72.0 and 1.2 to 73.0 during the kharif and rabi seasons of 2018, respectively. The maximum percent yield loss was noticed in the HS (75.0 –89.4), while HR possessed the minimum (1.2–2.0). The methanolic leaf extracts of highly resistant and highly susceptible genotypes with essential controls were subjected to gas chromatography–mass spectrometry analysis. Differential accumulation of metabolites was noticed, and a total of 81 metabolites representing 26 functional groups were identified. Both highly resistant and susceptible genotypes harbored eight unique classes, while ten biomolecules were common. The hierarchical cluster analysis indicated a distinct metabolite profile. Fold change in the common metabolites revealed an enhanced accumulation of sugars, alkanes, and carboxylic acids in the highly resistant genotype. The principal component analysis plots explained 93.7% of the variation. The metabolite profile showed a significant accumulation of three anti-viral (octadecanoic acid, diphenyl sulfone, and 2-Aminooxazole), one insecticidal (9,10-Secocholesta-5,7,10(19)-triene-3,24,25-triol), one antifeedant (cucurbitacin B), and six metabolites with unknown biological function in the highly resistant genotype.

## 1. Introduction

Legumes are an excellent rotational crop [1], and their cultivation enhances soil carbon levels, reduces the need for fossil fuel use in farming [2], reduces soil erosion, and improves soil fertility through symbiotic relationships [1]. The demand-driven changes in agriculture cultivation practices, such as intensification systems and unbalanced animal-food-based protein consumption, warrant the promotion of legume proteins [3]. Legumes adequately satisfy human protein requirements [4] and provide a balanced diet, as they possess a good quantity of fiber, carbohydrates, vitamins, and minerals [5,6]. The yield of legumes is relatively variable and low due to varied cultivable environments and susceptibility to pests and diseases. Horse gram (*Macrotyloma uniflorum* (Lam.) Verdc.) is a multipurpose diploid legume (2n = 20, 22 & 24) grown for seed and fodder purposes in the arid regions of Asia, Africa, and Australia [7]. The nutritional benefits of horse gram include a significant quantity of proteins (16.9%–30.4%) [8], lysine [9], phosphorus, iron, and vitamins [10]. Moreover, horse gram regulates oxygen-carrying capacity and calcium uptake [11]. Its therapeutic properties include treating edema, piles, renal stones, red blood cell agglutination, and anti-urolithiasis activity [12].

Horse gram is an annual, photosensitive, slender, twining herb with cylindrical tomentose stems. Due to its weak stem, horse gram cannot grow straight. Thus, it possesses a prostrating growth habit, producing a dense leaf mat covering at least 30–60 cm in land area. Further, the present-day horse gram varieties have an indeterminate growth habit that causes the crop to produce new leaves up until harvest. The crop grows in the field for at least 120 days from sowing (October) to harvest (February), facing two different seasons (winter and summer). During these growing seasons, the dense mat of leaves is affected by fewer foliar diseases, resulting in yield reduction [13]. Of these, the horse gram yellow mosaic virus (HgYMV) is a dreaded disease, causing complete crop failure. The HgYMV is a distinctive bipartite genomic begomovirus [14,15] transmitted by whitefly. The quantum of monetary loss concerning HgYMV in horse gram has not been reported because it is an under-exploited crop. However, the quantum of yield loss is significant when HgYMV infects the crop at the early stages of growth. Infection adversely affects the leaf size and growth, with a pronounced effect on seed color, texture, and size. Bashir et al. [16] reported a 10%–100% yield due to HgYMV infection, which has become grave in southern India.

The magnitude of the prevalence and severity of the YMV disease may be attributed to the availability of alternate hosts, vector populations, and weather parameters. With the severe incidence of YMV in winter pulse crops [17], the summer incidence of YMV is changing. Moreover, the incidence, spread, and management of the HgYMV disease depend on the vector population [18]. This continuum of inoculum and sufficient vector population threatens cultivation [19]. The application of pesticides could control the whitefly population and thereby contain the disease spread; however, it does not provide effective control.

Identification of HgYMV tolerant genotypes and their exploitation is the need of the hour. Similarly to other pulses, targeted systematic evaluation of horse gram germplasm could help in this quest, as it is a more efficient, eco-friendly, safe, and long-term solution [20]. Field screening for HgYMV under in vivo (may please be italicized) conditions has helped to identify the tolerant genotypes [21,22]. Percent disease index (PDI) and scaling methodology are the methods used for virus resistance screening [23]. Metabolomic analyses through gas chromatography–mass spectrometry (GC/MS) could help to understand the involvement of biomolecules in imparting resistance. Principal component analysis (PCA) was carried out in one study to reduce the complexity of the GC/MS data [24]. The YMV infection modified the vital metabolic processes. Differences in the accumulation of γ-amino butyric acid (GABA), sucrose, alanine, proline, tryptophan, phenylalanine, citrates, pyruvate, and ascorbate were observed [25].

The concept of a hierarchal-cluster-analysis- (HCA) based heat map helps to understand the diversity of compounds produced [26] under stress. Therefore, the present study aimed to screen the horse gram germplasm for HgYMV tolerance tagging extreme classes of resistance, to develop mapping populations, and to understand the metabolomics of viral infection. The metabolomic analysis would help in identifying functional metabolites exhibiting defense mechanisms during YMV development. These metabolites could be a valuable target for a better understanding of host-–pathogen (virus) interaction.

## 2. Materials and Methods

### 2.1. Plant Materials and Screening for HgYMV Resistance

A sample of a horse gram germplasm collection (252 genotypes) conserved at Dr. Ramaih gene bank, Tamil Nadu Agricultural University (TNAU), Tamil Nadu, India was utilized for screening against HgYMV at the Department of Pulses (Lat: 11.0238°, Long: 76.9279°, and Alt: 338.83 m), Centre for Plant Breeding and Genetics, TNAU, during rabi season (October to December) in 2017. The study comprised 252 genotypes, which include 250 local landraces and two popular cultivars (PAIYUR 2 and CRIDA 1-18 R). In the field, the genotypes were sown in four replications with a row length of 5 m. The spacing was 30 × 15 cm. Each replication comprised 66 plants per genotype. The susceptible genotype HG 22 [17] was grown after every five tester rows to ensure an adequate whitefly population, thereby ensuring disease spread. All the recommended cultivation practices were adopted *in toto,* except herbicide and pesticide sprays. Only manual weeding was done. Pesticide spray was avoided to ensure a sufficient whitefly population, thereby preventing false escapes. The crop was irrigated at regular intervals to ensure adequate lush leaf growth. The whitefly population was counted at weekly intervals in ten randomly selected plants per replication per genotype, and periodically monitored for the development of disease symptoms. A total of 266 plants grown in four replications for a genotype were considered for disease scoring.

### 2.2. Grouping of Horse Gram Genotypes

HgYMV tolerant genotypes were identified using the disease rating scale based on the host-plant resistance screening procedure followed in the All India Coordinated Research Project on MULLaRP. The HgYMV severity was recorded on a row basis using a 0–9 modified scale (Table 1) [27].

The observation on disease rating was used to calculate the Percent disease index, i.e., PDI = [sum of all the numerical ratings/(number of observations × maximum disease rating)] × 100 [28]. Subsequently, the horse gram germplasm was grouped into different categories based on host-plant resistance to HgYMV (Table 2).

The disease reactions of the genotypes of the extreme classes (highly resistant and highly susceptible) and the top three genotypes from moderately resistant, moderately susceptible, and susceptible classes were reconfirmed during the kharif (June–July) and rabi seasons of 2018. The number of genotypes was restricted in the confirmative studies to select highly promising genotypes for developing the mapping population for further exploitation. The kharif 2018 trial was laid out only in the field conditions (because the kharif season crop does not set pods), while in the rabi season 2018 experiment, the selected genotypes in each category, along with checks, were grown in field and pots in four replications to estimate the percent yield loss (PYL). The procedures of HgYMV scoring and whitefly count were adopted as done in the preliminary screening. The potted genotypes maintained in the net house served as control and were used for calculating yield loss due to HgYMV infection. The present disease index ranged from 1.2 to 72.0 and 1.2 to 73.0 during the kharif and rabi seasons of 2018, respectively. The maximum percent yield loss was noticed in the HS (75.0–89.4), while HR possessed the minimum (1.2–2.0). These results helped in tagging genotypes with extreme levels of resistance and susceptibility.

### 2.3. Confirmation of HgYMV Infection

During the experiments, DNA from the check, resistant, and susceptible accessions were extracted by the 2% CTAB method [29]. The quality of DNA was checked by a nanodrop spectrophotometer (Nanodrop 2000, Thermofisher, Waltham, MA, USA) and diluted to the required concentration. Primary YMV confirmation was carried out using degenerate Rojas primer [30], which resulted in an amplicon size of 1.2 kb. PCR products amplified by Rojas primer were partially sequenced by Sanger sequencing [31] and blasted against the NCBI (www.ncbi.nlm.nih (accessed on 17 December 2017)) database, which matched with the HgYMV DNA A component. A specific primer for diagnosing HgYMV was synthesized and utilized for confirmation in different experiments. The specific primer sequence (5′–3′) was ATCATACTGAGAACGCTTTG (forward) and TGTCATACTTCGCAGCTTC (reverse). The target region was the complete genome of HgYMV DNA A with an amplicon size of 2.7 kb. The polymerase chain reactions were set for 35 cycles with the temperature profiles as initial denaturation: 94 °C; denaturation: 94 °C; annealing: 55 °C; elongation: 72 °C and final elongation: 72 °C [15]. The delineated, highly resistant, and highly susceptible genotypes were utilized for metabolome analysis using GC/MS.

### 2.4. Metabolome Analysis

#### Preparation of Sample and Extraction of Metabolites

Fresh leaf samples from resistant and highly susceptible accessions and the respective controls were collected in three biological replicates and shade-dried for three days at room temperature. The dried leaf samples were powdered and subjected to extraction. The polar metabolites were extracted using 100% methanol (350 µL). Leaf samples (40 mg) were ground in liquid nitrogen and extracted with methanol. Further, it was suspended in internal polar standard, Ribitol (50 µL, 0.2 mg/mL in water) [32], incubated at 70 °C for 15 min, and mixed with an equal volume of distilled water. Chloroform (300 µL) was added to this mixture and centrifuged at 14,000 rpm for 10 min to separate the polar and non-polar metabolites. The supernatant was washed with chloroform. The polar phase aliquot (100 µL) was used to analyze the abundance of metabolites, and the non-polar phase was discarded. The aliquot was vacuum-dried, redissolved, and derivatized at 37 °C for 2 h. Methoxy-amine hydrochloride (40 µL of 30 mg/mL in pyridine) was used for derivatization. For trimethylsilylation, N-methyl-N-[trimethylsilyl] trifluoroacetamide (70 µL; MSTFA) was used at 37 °C for 30 min [33]. Merck chemicals were utilized for extraction (Kenilworth, NJ, USA)

### 2.5. GC/MS Analysis

The biochemical compounds present in the crude extract were detected by injecting 1 µL of the samples into a GC injection port (AI3000 II, Thermo Fischer Scientific, Waltham, MA, USA) connected to a GC/MS (TRACE™ GC Ultra with *DSQII* Quadrupole mass spectrometer (Thermo Fischer Scientific, Waltham, MA, USA). The system is equipped with an Agilent DB-5MS column of 30 m × 0.25 mm × 0.25 µm (length × diameter × film thickness). The carrier gas was helium, and the flow rate was 1 mL/min. Initially, the temperature was maintained at 150 °C with an increasing rate of 4 °C/min and finally elevated to 250 °C with a heating rate and holding time of 5 °C/min and 5 min, respectively. High-energy electrons were utilized in the ionization system under spectroscopic detection in GC/MS.

The split injection technique with a ratio of 1:10 was followed to prevent the overloaded peaks. The biochemical compounds involved in YMV susceptibility/resistance disease reactions were identified with the help of retention time. Retention time in GC-MS depicts the time for a compound to pass through the chromatography column. The pseudo peaks caused due to the internal standards or to the noise, column, and derivatization procedures were removed from the dataset. AMDIS (Automated Mass Spectral Deconvolution and Identification System Program) was used to extract the baseline corrected mass spectra of GC/MS output, and each peak’s retention time was identified. Using the retention time, the mass spectral fragments of each peak were manually checked for their consistency. The similarity index guidelines issued by NIST were followed to identify the metabolites. The MSTs (Mass Spectral Tags) of the peaks from all four replications were compared with the MSTs of metabolites showing the five best matches in the NIST and Golms Metabolome Database (http://csbdb.mpimp-olm.mpg.de/csbdb/gmd/gmd.html (accessed on 19 January 2018). The metabolite name was assigned based on the best spectral match [34]. For identifying significant metabolites, only the peaks with a similarity index higher than 70% were considered in the study. The match factor (SI) or reverse match factor (RSI) was utilized to assess the goodness of fit of an identified spectrum with the library reference. The spectra higher than 900 SI or RSI were grouped as excellent, 800–900 as good, 700–800 as fair, and below 600 as poor.

### 2.6. PCA and HCA Analysis

GC-MS data generated from leaf samples of resistant and susceptible genotypes were subjected to PCA analysis using R (https://www.R-project.org/ (accessed on 5 September 2019), R Core Team 2017). The corrected area and fold change were used for the analysis. PermuMatrix software Version 1.9.3 EN (available online at http://www.atgc-montpellier.fr/permutmatrix/) was used for the HCA (accessed on 5 September 2019), and the dissimilarity was measured based on Euclidean distance. The cluster was generated using the UPGMA method and represented as a heatmap.

## 3. Results

A selection of horse gram landraces at Tamil Nadu Agricultural University, India, were primarily screened for HgYMV tolerance during the rabi season of 2017 at the Coimbatore location. The host-plant resistance levels of the identified extreme classes were reconfirmed during the subsequent kharif and rabi seasons of 2018 at the Melalathur location, where the temperature was sufficient to harbor a sufficient whitefly population. The average whitefly count ranged from 15–21 and 18–26 during the rabi and kharif seasons, respectively, and these counts were found sufficient for the spread of HgYMV. These multilocation and seasonal experiments were conducted to avoid false escapes and identify novel genotypes/genes for further exploitation in the targeted breeding programs.

### 3.1. HgYMV Infection and Symptom Development

**Figure 1:**
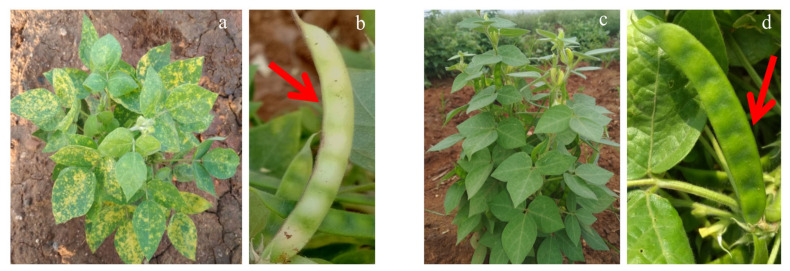
(**a**) A HgYMV infected plant of highly susceptible horse gram genotype PLS 6194. (**b**) A HgYMV infected yellow and ill-filled pod (indicated by red arrow) of the highly susceptible horse gram genotype PLS 6194. (**c**) A plant of highly resistant horse gram genotype PLS 6002 (**d**) A green and normal pod (indicated by red arrow) of the highly resistant horse gram genotype PLS 6002.

The developed genotypes showed no disease symptoms in all three experimental seasons until 14 days after sowing. After that, characteristic HgYMV symptoms slowly appeared, and the degree and severity of symptoms varied according to the host-plant resistance level. The symptoms first appeared in the young leaves as tiny yellow flecks. Consequently, the freshly emerging leaves exhibited more conspicuous and irregular alternate green and yellow patches. In the highly susceptible genotype, affected plants produced fewer small and malformed pods, and yellow spots were also observed on such deformed pods and seed coat. The seeds were small and pale in color. On the contrary, no symptoms or very few tiny yellow specks with restricted spread were noticed in the highly resistant genotype (Figure 1).

### 3.2. Confirmation of HgYMV Infection

**Figure 2:**
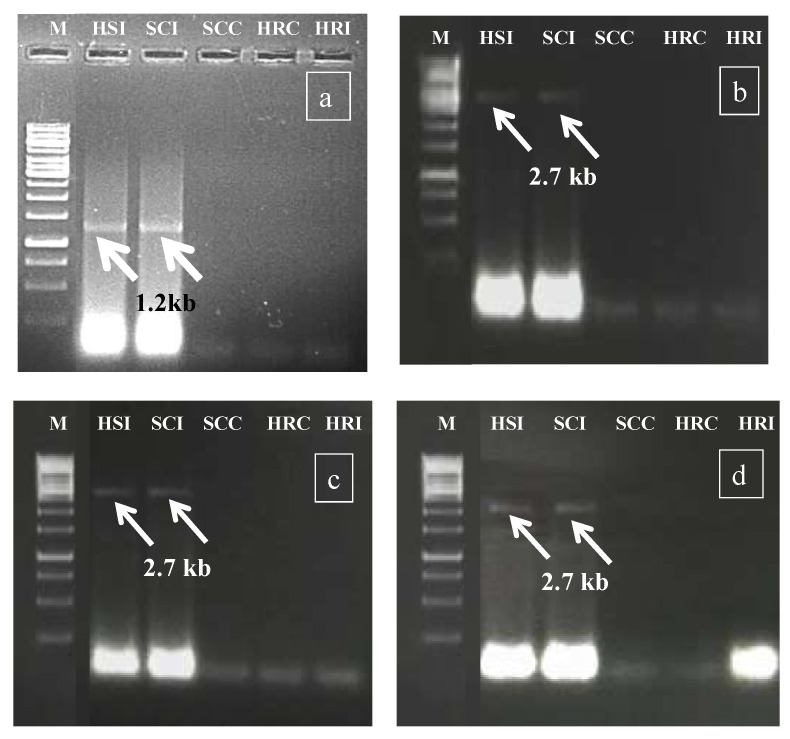
(**a**) Preliminary confirmation of Geminivirus infection by Rojas primer during rabi 2017. (**b**) Confirmation of HgYMV infection by HgYMV DNA A primer during rabi 2017. (**c**) Confirmation of HgYMV infection by HgYMV DNA A primer during kharif 2018. (**d**) Confirmation of HgYMV infection by HgYMV DNA A primer during rabi 2018. M—1 kb marker; HSI—highly susceptible infected; SCI—susceptible check infected; SCC—susceptible check control; HRC—highly resistant control; HRI—highly resistant infected.

During rabi 2017, in the preliminary screening experiment, the HgYMV infection was validated (Figure 2a) primarily by Rojas primer [30], followed by amplicon sequencing, NCBI blasting, HgYMV-specific primer synthesis, and confirmation (Figure 2b). In the subsequent seasons, the specific primer was used for HgYMV confirmation (kharif 2018: Figure 2c and rabi 208: Figure 2d). Across the experiments, in the susceptible check infected and highly susceptible genotype, an HgYMV-specific amplicon with a size of 2.7 kb was amplified, which was otherwise not amplified in the resistant genotypes (Figure 2c,d).

### 3.3. Genotype Categorization and Confirmation of HgYMV

The in vivo screening yielded different groups of genotypes. The percent disease index in the preliminary screening at the Coimbatore location varied between 1.42 (PLS6002) to 75.00% (PLS6194) (Table S1).

Based on the PDI and disease rating scale, the test genotypes were classified into 1 highly resistant (HR) (PLS6002), 35 moderately resistant (MR), 98 moderately susceptible (MS), 117 susceptible genotypes (MS), and 1 highly susceptible (HS) (PLS6194). The genotypes that developed infective symptoms in the early stages of growth were considered susceptible and highly susceptible. The host-plant resistance levels in the identified highly resistant and highly susceptible genotypes and the top three from the other groups were reconfirmed during the subsequent two seasons at the Melalathur location (Lat: 12.9196°, Long: 78.8734°, and Alt: 182 m), where the average temperatures were adequate to support a sufficient whitefly population. The promising genotypes were alone selected from each category for the confirmation study to select ideal genotypes for the development of trait-specific mapping populations in the future.

In the confirmatory trials, no deviation was observed in the host-plant resistance levels of the genotypes, thus affirming the findings of the preliminary HgYMV screening and categorization findings. No significant difference was observed across two seasons for PDI in the genotypes of HR, MR, and MRC classes. However, a slight increase in PDI was observed in the rabi season in the MS, S, and HS genotypes, indicating a link between genotype and HgYMV vulnerability. The checks maintained the PDI across seasons. The maximum PYL was noticed in the HS (75.0–89.4), while HR possessed the minimum (1.2 –2.0) (Table 2). Therefore, these two extreme classes of genotypes were considered for metabolomic studies through GC/MS to understand the role of biomolecules in host-plant resistance with respect to HgYMV infection.

### 3.4. GC–MS Chromatography

**Figure 3:**
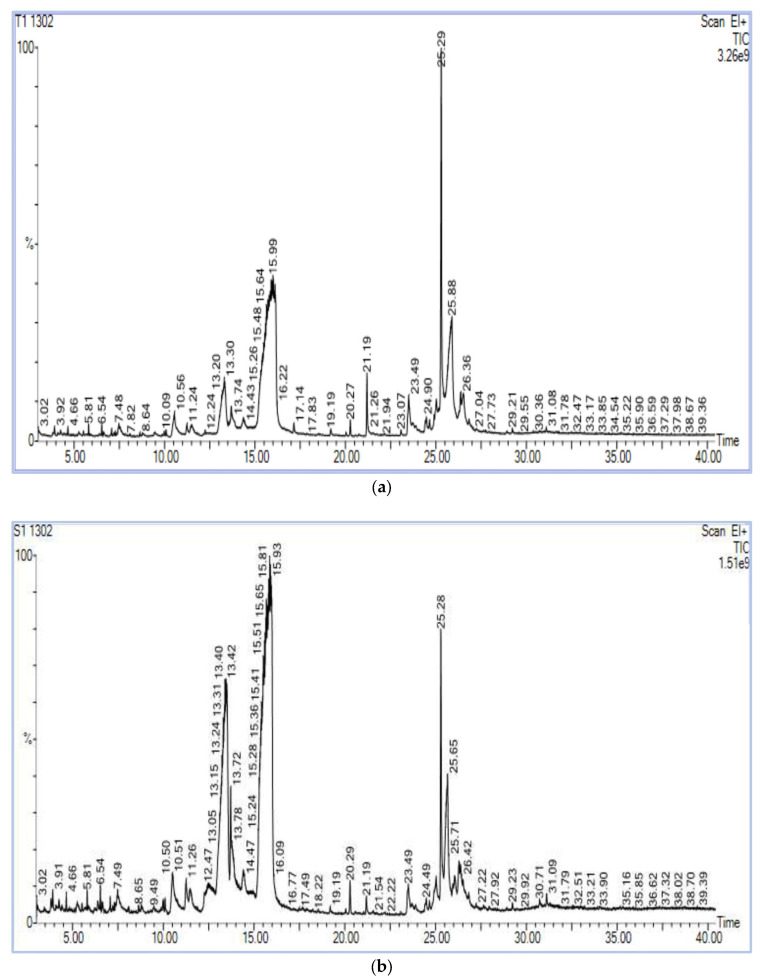
(**a**) Chromatogram of methanol extract of HgYMV highly resistant genotype. (**b**) Chromatogram of methanol extract of HgYMV highly susceptible genotype.

The chromatograms of the samples produced a strong signal, and portrayed a larger peak capacity and consistent retention time (Figure 3), indicating the dependability of the present metabolomic analysis.

A total of 81 metabolites representing 26 functional groups were identified. Of them, alcohol, alkane, carboxylic acid, and sugars were expressed in HS, HR, and the respective checks, indicating their multiple functions. HgYMV infection triggered the accumulation of thirteen and twelve classes of compounds in HS and HR, respectively, which were otherwise not expressed in the respective controls. A common accumulation of a few biomolecules, such as glycoside, in the susceptible group and benzene and ketone in the resistant group was observed between test entries and respective checks. However, the quantum of accumulation was high in HS and HR (Table 3).

**Figure 4:**
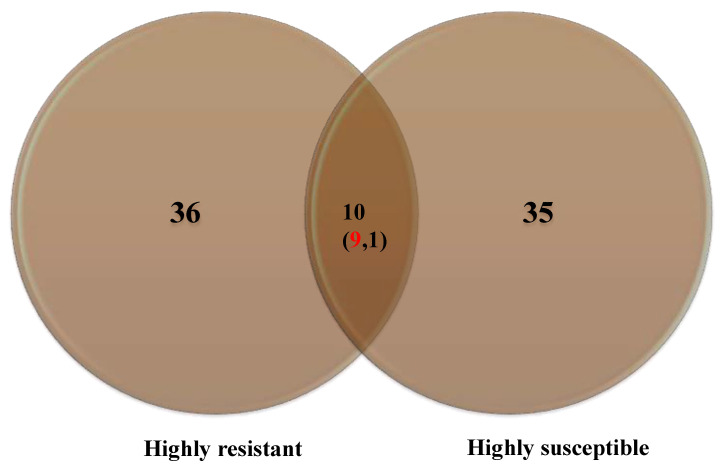
Number of metabolites accumulated in the highly resistant and highly susceptible genotypes. Numbers in the parenthesis indicate upregulated (red) and down-regulated metabolites (black).

A maximum number of metabolites (36) were identified in HR compared to HS (35), with ten common metabolites (Figure 4).

The notably expressed compounds in HR were sugars (59.226%), alkane (12.981%), glycoside (6.277%), carboxylic acid (3.658%), aldehyde (1.993%), ketone (1.991%), alkene (0.667%), and alcohol (0.661%). Conversely, a diverse accumulation spectrum of compounds was observed in HS in the following order: sugar (53.648%), glycoside (17.941%), alkane (8.0345%), carboxylic acid (2.421%), ketone (2.381%), alcohol (1.156%), and cyclic carboxylic ester (0.766%) (Table 3). The virtue of the fitness of biomolecules with the NIST library is furnished in Table 4.

A total of eleven good and nine fair matches were identified in HS. Similarly, eleven good and seven fair matches were observed in HR. The unique molecules identified in HR were amine, amino acid, aromatic oxazole, azide, glucosinolate, heterocyclic organo-oxygen compound, isoquinoline, and lanostane skeleton. In HS, amide, cyclic azine, cyclic carboxylic ester, ester, ether, heterocyclic dioxygen compound, and phosphonic acid were the unique molecules (Table 5).

**Figure 5:**
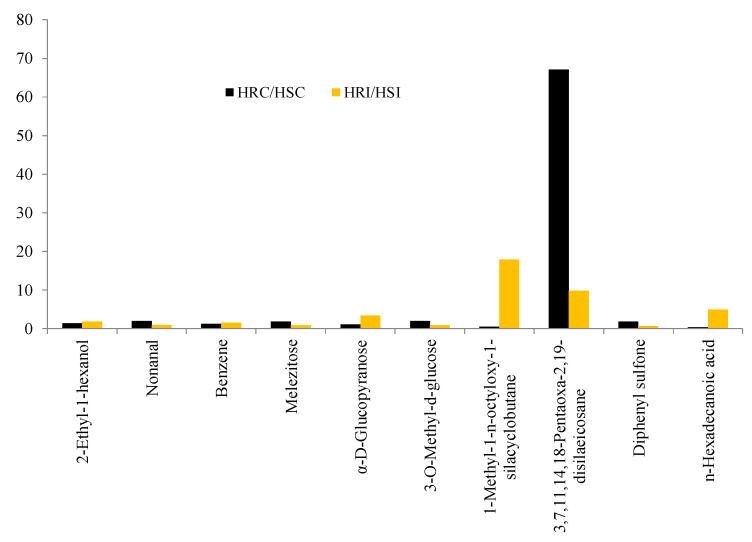
Fold change analysis of common metabolites.

Metabolites such as 2-Ethyl-1-hexanol, Nonanal, Benzene, Melezitose, α-D-Glucopyranose, 3-O-Methyl-d-glucose, 1-Methyl-1-n-octyloxy-1-silacyclobutane, 3,7,11,14,18-Pentaoxa-2,19-disilaeicosane, Diphenyl sulfone, and n-Hexadecanoic acid were commonly expressed in both HR and HS genotypes (Table 5). However, the accumulation level was varied, and the fold increase is presented in Figure 5.

Accumulation was significant for 1-Methyl-1-n-octyloxy-1-silacyclobutane (18-fold), 3,7,11,14,18-Pentaoxa-2,19-disilaeicosane (10-fold), and n-Hexadecanoic acid (5-fold) in HR under infected conditions.

### 3.5. PCA and HCA

**Figure 6:**
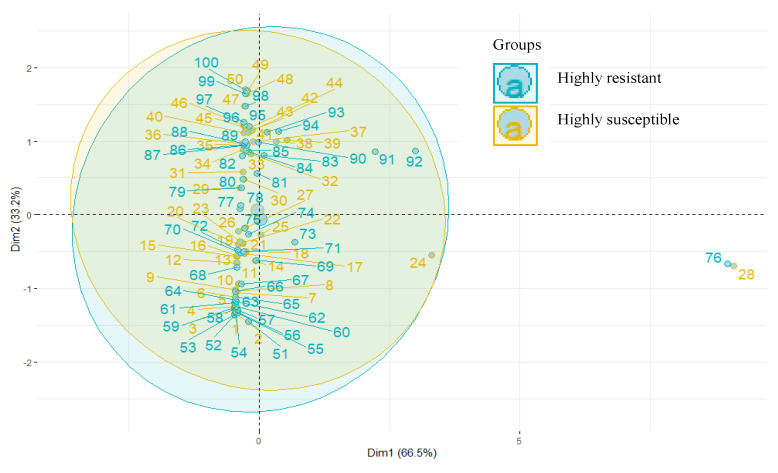
Principal component analysis of metabolites.

The ten common metabolites identified in HR and HS were subjected to PCA, and an apparent separation was noticed (Figure 6).

**Figure 7:**
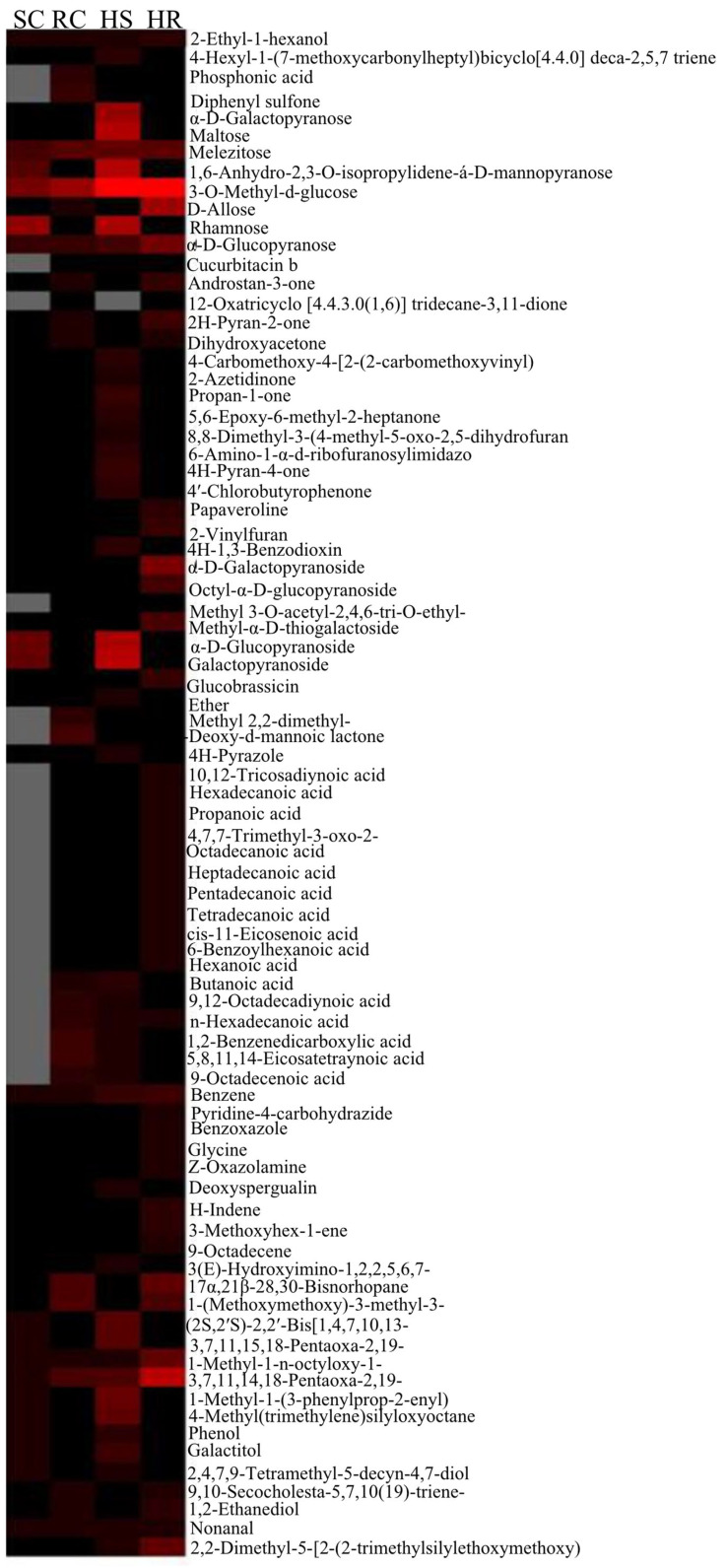
Hierarchical cluster analysis of metabolites accumulated in different resistant and susceptible genotypes. Colors in the heat map indicate the total content of the metabolite.

The first two PCs were plotted together, explaining 93.7% of the total variation. HCA indicated a distinct metabolite profile in both genotypes. The heat map depicted a differential accumulation of sugars, alcohol, ketones, carboxylic acid, glycosides, and alkanes (Figure 7).

## 4. Discussion

Horse gram is a legume of tropics and subtropics grown mostly as a food crop in larger areas during the rabi season in the marginal lands as a rainfed crop. Horse gram is exposed to winter extremes, thereby being affected by a few foliar diseases, such as HgYMV, causing an alarming yield loss [13]. Earlier, Prema and Rangaswamy [35] reported that HgYMV initially caused leaf yellow discoloration followed by the development of greenish yellow mosaic symptoms; thereon, reduction in leaf size, stunted plant growth, and yield loss was witnessed.

### 4.1. HgYMV Screening, Categorization of Genotypes, and Confirmative Studies

During the preliminary HgYMV resistance screening (rabi 2017), the genotypes were free from symptom development for two weeks after sowing; thereon, based on the host-plant resistance level characteristic, mosaic symptom development was witnessed. The HgYMV infection was confirmed by specific primers during the experimental years. In the preliminary screening, most of the genotypes succumbed to HgYMV and were classified as S and MS. Interestingly, two extreme classes of genotypes, HR (PLS6002) and HS (PLS6194), were also identified. These two genotypes and three top promising genotypes from the classes MR, MS, and S, with respective checks, were considered for the confirmative trials (kharif and rabi seasons, 2018). During these studies, the HgYMV infection was confirmed using HgYMV-specific amplicon. These trials confirmed the results of preliminary screening for genotype categorization. The genotypes of varied resistant classes maintained the PDI, while an increase was witnessed in susceptible genotypes.

### 4.2. GC/MS Analysis, PCA, and HCA

The extreme classes of genotypes (HR and HS) were utilized for the metabolome analysis through GC/MS to understand the biomolecule synthesis/activation in response to HgYMV infection in horse gram. HgYMV infection induced a wide spectrum of biomolecules. The total and unique biomolecules were high in the HR. A total of ten common metabolites were expressed in HR and HS genotypes. The expressed quantity of common metabolites was higher in the HR than in the HS under infected conditions. Of them, accumulation was significant for 1-Methyl-1-n-octyloxy-1-silacyclobutane, 3,7,11,14,18-Pentaoxa-2,19-disilaeicosane, and n-Hexadecanoic acid indicating their role in disease resistance. However, in the uninfected condition, significance was noticed only for 3,7,11,14,18-Pentaoxa-2,19-disilaeicosane. A higher level of 3,7,11,14,18-Pentaoxa-2,19-disilaeicosane was detected in HR under infected and uninfected conditions, indicating that its synthesis is specific to the genotype. These ten common metabolites were alone considered for PCA and HCA analyses to understand the significance of accumulation concerning HgYMV resistance/susceptibility. The PCA separated the common metabolites, apparently, and the maximum variation was explained by the first two PCs. The HCA portrayed a distinctive metabolite profile between HR and HS, and a differential accumulation of metabolites was noticed. Earlier, the complexity of GC/MS data was simplified by PCA [24], and HCA was used to understand the diversity pattern of compounds [26]. A detailed narration of the roles of varied biomolecules expressed due to HgYMV infection helps identify resistant/susceptible linked markers that can be utilized to accelerate the targeted breeding programs.

### 4.3. The Biological Significance of Biomolecules Accumulated in the HR Genotype PLS6002

#### 4.3.1. Unique Biomolecules

Accumulation of a few unique biomolecules cucurbitacin B, glycine, vinyl furan, 1,2-Ethanediol, α-D-galactopyranoside, glucobrassicin, 2H-pyran-2-one, diphenyl sulfone and papaveroline was observed. Cucurbitacin B is a bitter-tasting secondary metabolite of Cucurbitaceae plants, predominantly produced in fruits and not transported to other plant parts [36]. It acts as an antifeedant (kairomones) through Cuc receptors of maxillary palpi [37]. HgYMV is transmitted by whitefly; therefore, it is presumed that cucurbitacin B might have influenced the feeding behavior of whitefly in HR. However, this needs further confirmatory studies. In the current study, two new observations made concerning cucurbitacin are (i) its presence in legume plants and (ii) its presence in leaf extracts.

Glycine plays an important role in maintaining the intracellular concentration of one-carbon groups and modulates the transmembranous trafficking of Ca^++^. Ionized calcium is a pivotal element in cell signal transduction and acts as a cytoprotectant [38]. Glycine also participates in the formation of glycine-rich proteins, whose expression is regulated by external stress stimulus. Therefore, the accumulation of glycine may have helped in effective signal transduction in HR, thereby conferring resistance.

Furan derivatives possess antimicrobial activity [39]. Vinyl furan chemically modifies the function of sulfhydryl groups of thiol enzymes and thereby affects the energy metabolism of pathogens. The antibacterial activity of 1,2-Ethanediol (ethylene glycol) is well documented [40]. The HR produced glycosides (α-D-Galactopyranoside) and glucosinolates (glucobrassicin), which are activated upon pathogen infestation [41]. The rapid hydrolysis of glucosinolate produces isothiocyanates (ITCs) [42] that restrict the pathogen’s growth [43].

The 2H-Pyran-2-one (2-pyrone) derivatives displayed antimicrobial activities [44] and served as a building block for heterocycle biosynthesis. The antimicrobial activities of sulfones are reported [45]. Heterocyclic compounds with diphenyl sulfone moiety exhibited a significant improvement in the antiviral and other defensive properties of HR [46]. The antimicrobial and antioxidant activities of papaveroline and 2,2-Dimethyl-5-[2-(2-trimethylsilyl ethoxy methoxy)-propyl]-[1,3]dioxolane-4-carboxaldehyde were documented by [47,48], respectively.

The oxazole group derivatives, such as 2-Oxazolamine or 2-Aminooxazole, exhibit antiviral and fungicidal properties [49]. The biomolecule 9,10-Secocholesta-5,7,10(19)-triene-3,24,25-triol inhibited the activity of the adult cowpea storage pest, *Callosobruchus maculatus* [50]. Accumulation of this biomolecule has a dual advantage of insecticidal properties against whitefly in the field and beetle at storage. It is assumed to be significant, as horse gram is a one-season crop that requires long-term storage before marketing and consumption.

#### 4.3.2. Other Significant Biomolecules: Sugars, Alkanes and Carboxylic Acids

**Figure 8:**
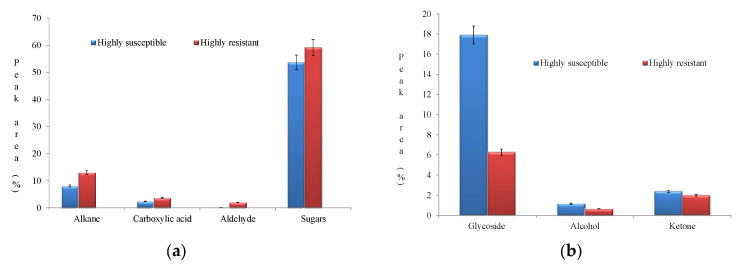
(**a**) Enhanced accumulation pattern of biomolecules in the highly resistant genotype. (**b**) Enhanced accumulation pattern of biomolecules in the highly susceptible genotype.

Upon pathogen infestation, plants activate several defense mechanisms to counteract the pathogen’s virulence. These responses upregulate several biomolecules from different metabolic pathways. In HR, enhanced accumulation of sugars, alkanes, and carboxylic acid were observed (Figure 8a).

Sugars act as signaling molecules in host-pathogen interaction [51,52] and immunity [53]. HgYMV induced the accumulation of a rare sugar, D-Allose, which upregulates many defense-related PR-protein and associated genes [54,55].

Plant cells have cuticle coating on the outer surface of epidermal cells, providing tolerance against various biotic and abiotic stresses. The cuticle is a combination of cutin and cuticular wax. The cuticular wax is made of a mixture of very long-chain fatty acids (C20–C40). Stresses induce the production of cuticular wax derivatives, such as alkanes, aldehydes, primary and secondary alcohols, ketones, esters, and secondary metabolites [56]. In HR, an over-accumulation of alkanes was noticed. This may have protected the cell wall from virus-induced damages, thereby conferring resistance.

Carboxylic acid regulates the transcription of sugar signaling pathways, and thus confers resistance against biotic stress [57]. The HR exhibited upregulation of many carboxylic acid molecules, *viz.*, 10, 12-tricosadiynoic acid, propanoic acid, 4,7,7-trimethyl-3-oxo-2-oxabicyclo[2.2.1] heptane1-carboxylic acid, pentadecanoic acid, tetradecanoic acid, n-hexadecanoic acid, octadecanoic acid, hexanoic acid (Hx), and cis-11-Eicosenoic acid. The defensive roles of 10, 12-tricosadiynoic acid and propanoic acid were reported [58,59,60]. Kasuga et al. [61] reported significant antimicrobial activity of 4,7,7-trimethyl-3-oxo-2-oxabicyclo[2.2.1] heptane1-carboxylic acid (Camphanic acid). Pentadecanoic, tetradecanoic, and n-Hexadecanoic acid exhibited protective roles against pathogens [62,63]. Hx priming induced disease resistance against *Botrytis cinerea* [64]. Octadecanoic acid exhibited antiviral, antibacterial, and antioxidant activities [65]. Although the accumulation of cis-11-Eicosenoic acid was reported under salt stress [66], in our study, it was observed in response to HgYMV infection.

The higher accumulation of sugars, alkanes, and carboxylic acids, as well as an accrual of cucurbitacin B, glycine, vinyl furan, 1,2-ethanediol, α-D-galactopyranoside, glucobrassicin, 2H-pyran-2-one, diphenyl sulfone and papaveroline, helped PLS6002 to resist the pathogenicity of HgYMV, and thus emerged as a highly resistant genotype. A total of six new metabolites 12-Oxatricyclo [4.4.3.0(1,6)] tridecane-3,11-dione, 1-(Methoxymethoxy)-3-methyl-3-hydroxybutane, 3-Methoxyhex-1-ene, Pyridine-4-carbohydrazide, Androstan-3-one, and 17α,21β-28,30-Bisnorhopane were observed in the HR, whose biological activities are less understood and need further studies.

### 4.4. The Biological Significance of Accumulation of Biomolecules in the HS Genotype PLS6194

#### Ketones, Glycosides, and Alcohols

The metabolites ketones, glycosides, and alcohols were upregulated in HS (Figure 8b).

A sufficient quantity of ketones is required to maintain the signal transduction and functionality of mitochondria and protect tissues from free radical damage. Ketones exhibit antimicrobial activity by interfering with pathogens by inhibiting cell wall synthesis, nucleic acid, and protein synthesis, and metabolic pathways [67]. The antiviral properties of long-chain hydroxyl ketones have been reported [68].

Pathogen invasion activates glycosides through hydrolysis and releases sugar as a by-product. Broken sugar plays an essential role in signal transduction [52] and immunity triggering [53]. Glycoside is involved in the salicylic acid synthesis, acting as an antioxidant and herbivore deterrent. The deficits in sugar and glycoside may adversely affect the activation of defense cycles, signal communication processes, and membrane integrity.

The plant cell wall is assumed to be essential for developmental processes and acts as a physical barrier against pathogen invasion. During infections, pathogens produce several hydrolytic enzymes to collapse the cell wall integrity [69], activating plant pectin methylesterases (PMEs). Plant PMEs play a significant role in developmental processes and help to maintain cell wall integrity against pathogen infection. These cell wall modifications release oligogalacturonide fragments (OGAs) and methanol. OGAs and methanol activate signaling and several defense genes, thereby conferring resistance.

Although the stress-responsive metabolites, such as ketones, alcohols, and glycosides, were upregulated in HS, they failed to resist the pathogen invasion and developed characteristic HgYMV symptoms. The stress-responsive function in HS may not be functional, and/or some of the metabolites might be diverted for the plant developmental process to enable it to survive in the stressed situation, evidenced by a few poorly filled pods and new flowers occurring up until harvest.

## 5. Conclusions

The in vivo HgYMV screening yielded different host-plant resistance classes of horse gram genotypes, and these classes maintained the PDI across seasons. A relationship between genotype and HgYMV severity was established. A spectrum of biomolecules responsible for HgYMV resistance was identified through GC/MS. The HCA analysis revealed the accumulation of a distinct metabolite profile in HR and HS genotypes. Significant upregulated synthesis of biomolecules of varied pathways, such as secondary metabolite, signal transduction, glucosinolate, heterocycle biosynthesis, oxazole, and fatty acid, enabled HR to resist HgYMV infection with the least PYL. A total of six newer metabolites, 12-Oxatricyclo [4.4.3.0(1,6)] tridecane-3,11-dione, 1-(Methoxymethoxy)-3-methyl-3-hydroxybutane, 3-Methoxyhex-1-ene, Pyridine-4-carbohydrazide, Androstan-3-one, and 17α,21β-28,30-Bisnorhopane were identified only in HR, which needs to be explored. Although ketones, alcohols, and glycosides were upregulated in the HS, it failed to resist the pathogenicity due to its photosynthate diversification for developmental processes instead of defensive roles.

## Figures and Tables

**Table 1:** Details of YMV screening scales.

Grade	Disease Symptom	Category
**0**	No plants showing symptoms.	Immune
**1**	No visible symptoms or minute yellow specks on leaves.	Free
**2**	Small yellow spots with restricted spread covering 0.1% to 5% leaf area.	Highly resistant (HR)
**3**	Mottling of leaves covering 6% to 10% leaf area.	Resistant (R)
**4**	Yellow of mottling leaves covering 11% to 15% leaf area.	Moderately resistant (MR)
**5**	Yellow of mottling leaves covering 15% to 20% leaf area.	Moderately susceptible (MS)
**6**	Yellow coloration of 21%–30% leaves and yellow pods.	Susceptible (S)
**7**	Pronounced yellow mottling and discoloration of leaves and pods, reduction in leaf size, and stunting of the plant covering 30% to 50% of the foliage.	
**8**	Severe yellow discoloration of entirety of leaves covering above 50% to 75% of foliage, stunting of plants, and reduction in pod formation.	Highly susceptible (HS)
**9**	Severe yellowing of leaves covering above foliage, stunting of plants, and no pod formation.	

**Table 2:** Details of host-plant resistance and yield loss consequent to HgYMV infection in horse gram.

S. No.	Genotype	Kharif 2018			Rabi 2018		
		PDI	Grade	Range of PYL *	PDI	Grade	Range of PYL
	Highly resistant (HR)						
1	PLS 6002	1.2	2	--	1.2	2	1.2–2.0
	Moderately resistant (MR)						
1	PLS6039	12.5	4	--	12.5	4	7.60–9.40
2	PLS6008	12.5	4	--	12.5	4	8.30–10.2
3	PLS6013	12.5	4	--	12.5	4	10.2–11.6
	Moderately susceptible (MS)						
1	PLS 6004	20.0	5	--	20.5	5	23.1–32.5
2	PLS 6183	19.0	5	--	19.5	5	32.3–35.6
3	PLS 6006	19.0	5	--	19.0	5	34.2–38.9
	Susceptible (S)						
1	2448984	49.0	7	--	49.5	7	42.1–46.5
2	PLS 6081	43.0	7	--	44.0	7	49.8–46.7
3	PLS 6046	42.0	7	--	43.0	7	53.5–61.8
	Highly susceptible (HS)						
1	PLS 6194	72.0	9	--	73.0	9	75.0–89.4
	Susceptible check (SC)						
1	HG 22: SC	49.0	7	--	49.0	7	46.9–62.7
	Moderately resistant checks (MRC)						
1	CRIDA 1-18R	12.50	4	--	12.50	4	12.8–12.4
2	PAIYUR 2	12.25	4	--	12.25	4	10.1–10.9

* Not calculated because horse gram can set seed only during rabi season. PDI: Percent disease index. PYL: Percent yield loss.

**Table 3:** List of differentially expressed biomolecules consequent to HgYMV infection in horse gram.

S. No	Class	Peak Area (%)			
		SC	HS	RC	HR
1	Alcohol	0.142	1.156	0.286	0.661
2	Aldehyde	0.002	0.175	0.004	1.993
3	Alkane	0.212	8.035	3.145	15.30
4	Alkene	-	0.164	-	0.667
5	Amide	-	0.144	-	-
6	Amine	-	-	-	0.161
7	Amino acids	-	-	-	0.151
8	Aromatic oxazole	-	-	-	0.163
9	Azide	-	-	-	0.146
10	Benzene	0.113	0.354	0.143	0.561
11	Carboxylic acid	0.684	2.421	0.268	3.658
12	Cyclic azines	-	0.119	-	-
13	Cyclic carboxylic ester	-	0.766	-	-
14	Ester	-	0.204	-	-
15	Ether	-	0.119	-	-
16	Glucosinolate	-	-	-	0.412
17	Glycoside	4.289	17.914	-	6.277
18	Hetero cyclic dioxin	-	0.209	-	-
19	Heterocyclic organo oxygen	-	-	-	0.406
20	Isoquinoline	-	-	-	0.203
21	Ketone	-	2.381	0.152	1.991
22	Lanostane skeleton	-	-	-	0.158
23	Sugars	21.124	53.648	22.684	59.226
24	Organo sulphur compound	-	0.393	-	0.274
25	Phosphonic acid	-	0.248	-	-
26	Triene	-	0.304	-	-

SC—susceptible check; HS—highly susceptible; HR—highly resistant; RC-resistant check.

**Table 4:** The goodness of fit of the extracted biomolecules from HgYMV HR and HS genotypes based on SI and RSI values.

S. No.	Class	Biomolecule	HS				HR			
			SI Value	RSI Value	RT	% Area	SI Value	RSI Value	RT	% Area
1	Alcohol	2-Ethyl-1-hexanol	813 *	899 *	4.664	0.125	786	886 *	4.664	0.227
		2,4,7,9-Tetramethyl-5-decyn-4,7-diol	747	818 *	10.106	0.151	-	-	-	-
		Galactitol	407	585	12.522	0.562	-	-	-	-
		Phenol	645	727 **			-	-	-	-
		9,10-Secocholesta-5,7,10(19)-triene-3,24,25-triol	-	-	-	-	439	485	3.599	0.163
		1,2-Ethanediol	-	-	-	-	557	834 *		
2	Aldehyde	Nonanal	801 *	857 *	5.814	0.171	806 *	849 *	5.810	0.169
		2,2-Dimethyl-5-[2-(2-trimethylsilylethoxymethoxy)-propyl]-[1,3]dioxolane-4-carboxaldehyde	-	-	-	-	632	675	23.492	1.824
3	Alkane	4-Methyl(trimethylene)silyloxyoctane	621	640	25.942	0.173	-	-	-	-
		1-Methyl-1-(3-phenylprop-2-enyl)oxy-1-silacyclobutane	431	539	12.692	0.299	-	-	-	-
		3,7,11,14,18-Pentaoxa-2,19-disilaeicosane	651	653	13.718	2.182	643	649	14.368	0.594
		1-Methyl-1-n-octyloxy-1-silacyclobutane	536	649	24.652	0.110	620	660	24.417	0.214
		3,7,11,15,18-Pentaoxa-2,19-disilaeicosane	641	667			-	-	-	-
		(2S,2′S)-2,2′-Bis[1,4,7,10,13-pentaoxacyclopentadecane]	466	597	31.284	0.110	-	-	-	-
		1-(Methoxymethoxy)-3-methyl-3-hydroxybutane	-	-	-	-	569	699	4.264	0.407
		17α,21β-28,30-Bisnorhopane	-	-	-	-	479	526	30.710	0.120
4	Alkene	3(E)-Hydroxyimino-1,2,2,5,6,7-hexamethylbicyclo[3.2.0]hept-6-ene	465	598	26.823	0.164	-	-	-	-
		9-Octadecene	-	-	-	-	464	493	3.694	0.134
		3-Methoxyhex-1-ene	-	-	-	-	560	761 **	4.469	0.136
		H-Indene	-	-	-	-	513	539	23.897	0.397
5	Amide	Deoxyspergualin	559	592	4.264	0.144	-	-	-	-
6	Amine	2-Oxazolamine	-	-	-	-	517	542	5.529	0.161
7	Amino acids	Glycine	-	-	-	-	406	453	11.627	0.151
8	Aromatic oxazole compound	Benzoxazole	-	-	-	-	603	846 *	4.584	0.163
9	Azide	Pyridine-4-carbohydrazide	-	-	-	-	690	712 **	6.540	0.146
10	Benzene	Benzene	678	821 *	7.490	0.354	684	805 *	7.480	0.561
		9-Octadecenoic acid	495	519	26.042	0.611	-	-	-	-
		5,8,11,14-Eicosatetraynoic acid	449	466	13.933	0.148	-	-	-	-
		1,2-Benzenedicarboxylic acid	599	827 *	19.195	0.108	-	-	-	-
		n-Hexadecanoic acid	755	817 *	21.191	0.272	857 *	866 *	21.186	1.342
		9,12-Octadecadiynoic acid	523	532	26.373	0.908	-	-	-	-
		Butanoic acid	614	794 **	30.714	0.258	-	-	-	-
		Hexanoic acid	-	-	-	-	578	747 **	3.829	0.263
		6-Benzoylhexanoic acid	-	-	-	-	544	647	5.274	0.169
11	Carboxylic acid	cis-11-Eicosenoic acid	-	-	-	-	487	548	9.486	0.142
		Tetradecanoic acid	-	-	-	-	723 **	776 **	17.144	0.205
		Pentadecanoic acid	-	-	-	-	625	688	19.195	0.175
		Heptadecanoic acid	-	-	-	-	686	736 **	23.072	0.120
		Octadecanoic acid	-	-	-	-	720 **	757 **	24.902	0.281
		4,7,7-Trimethyl-3-oxo-2-oxabicyclo[2.2.1]heptane-1-carboxylic acid	-	-	-	-	519	560	26.828	0.539
		Propanoic acid	-	-	-	-	479	513	26.968	0.157
		Hexadecanoic acid	-	-	-	-	492	507	27.238	0.123
		10,12-Tricosadiynoic acid	-	-	-	-	552	573	29.214	0.142
12	Cyclic azines	4H-Pyrazole	411	549	10.791	0.119	-	-	-	-
13	Cyclic carboxylic ester	3-Deoxy-d-mannoic lactone	622	749 **	14.423	0.766	-	-	-	-
14	Ester	Methyl 2,2-dimethyl-3,6,9,12,15,18,21-heptaoxa-2-silatricosan-23-oate	492	547	12.342	0.204	-	-	-	-
15	Ether	Ether	518	729 **	29.229	0.119	-	-	-	-
16	Glucosinolate	Glucobrassicin	-	-	-	-	490	498	24.472	0.412
17	Glycoside	Galactopyranoside	442	510	23.686	0.394	-	-	-	-
		α-D-Glucopyranoside	879 *	882 *	13.462	17.520	-	-	-	-
		Methyl-α-D-thiogalactoside	-	-	-	-	801 *	875 *	13.303	4.565
		Methyl 3-O-acetyl-2,4,6-tri-O-ethyl-α-d-mannopyranoside	-	-	-	-	628	665	13.698	0.904
		Octyl-α-D-glucopyranoside	-	-	-	-	464	539	23.707	0.579
		α-D-Galactopyranoside	-	-	-	-	552	572	24.652	0.229
18	Hetero cyclic dioxin	4H-1,3-Benzodioxin	708 **	741 **	6.540	0.209	-	-	-	-
19	Heterocyclic organo oxygen	2-Vinylfuran	-	-	-	-	777 **	852 *	3.919	0.406
20	Isoquinoline	Papaveroline	-	-	-	-	578	672	31.085	0.203
		4′-Chlorobutyrophenone	554	641	5.269	0.168	-	-	-	-
		4H-Pyran-4-one	714	882 *	6.395	0.201	-	-	-	-
		6-Amino-1-α-d-ribofuranosylimidazo[4,5-c]pyridin-4(5H)-one	474	527	6.680	0.117	-	-	-	-
		8,8-Dimethyl-3-(4-methyl-5-oxo-2,5-dihydrofuran-2-yloxymethylene)-3a,4,6,7,8,8b-hexahydro-3H-indeno[1,2-b]furan-2,5-dione	485	516	7.535	0.421	-	-	-	-
		5,6-Epoxy-6-methyl-2-heptanone	529	662	10.001	0.164	-	-	-	-
21	Ketone	Propan-1-one								
		2-Azetidinone								
		4-Carbomethoxy-4-[2-(2-carbomethoxyvinyl)benzyl]-3-methoxy-2,5-cyclohexadien-1-one								
		Dihydroxyacetone								
		2H-Pyran-2-one								
		12-Oxatricyclo [4.4.3.0(1,6)] tridecane-3,11-dione								
		Androstan-3-one								
22	Lanostane skeleton	Cucurbitacin b	-	-	-	-	413	476	16.614	0.158
23	Sugars	α-D-Glucopyranose	753	882 *	26.282	0.832	653	680	26.523	2.834
		Rhamnose	695	759 **	3.824	0.197	-	-	-	-
		D-Allose					722	826 *	11.527	0.619
		3-O-Methyl-d-glucose	753 **	768 **	15.863	44.605	742 **	765 **	15.994	38.406
		1,6-Anhydro-2,3-O-isopropylidene-α-D-mannopyranose	639	679	23.491	0.920	-	-	-	-
		Melezitose	729 **	731 **	10.521	1.813	658	673	35.883	14.636
		Maltose	675	687	25.647	4.051	-	-	-	-
		α-D-Galactopyranose	532	562	24.487	0.269	619	661	24.417	0.214
24	Organo sulphur compound	Diphenyl sulfone	827 *	862 *	20.285	0.393	799	831 *	20.270	0.274
25	Phosphonic acid	Phosphonic acid	789	830 *	3.914	0.248	-	-	-	-
26	Triene	4-Hexyl-1-(7-methoxycarbonylheptyl)bicyclo[4.4.0]deca-2,5,7-triene	452	496	26.618	0.304	-	-	-	-

RT—Retention time; HS—Highly susceptible; HR—Highly resistant; SI: Match factor; RSI: Reverse match factor; * Good (800–900 either SI or RSI); ** Fair( 700–800 either SI or RSI).

**Table 5:** Details of biomolecules expressed in horse gram in response to HgYMV infection.

S. No.	Class	Biomolecule	Chemical Formula	MW (g/mol)	HS	SC	HR	RC
1	Alcohol	2-Ethyl-1-hexanol	C_8_H_18_O	130.23	√	√	√	√
		2,4,7,9-Tetramethyl-5-decyn-4,7-diol	C_14_H_26_O_2_	226.35	√	√	-	-
		Galactitol	C_6_H_14_O_6_	182.17	√	√	-	-
		Phenol	C_6_H_6_O	94.11	√	√	-	-
		9,10-Secocholesta-5,7,10(19)-triene-3,24,25-triol	C_27_H_44_O_3_	416.60	-	-	√	√
		1,2-Ethanediol	(C_2_H_4_O)_n_H_2_O	62.07	√	√	√	√
2	Aldehyde	Nonanal	C_9_H_18_O	142.24	√	-	√	-
		2,2-Dimethyl-5-[2-(2-trimethylsilylethoxymethoxy)-propyl]-[1,3]dioxolane-4-carboxaldehyde	C_15_H_30_O_5_Si	318.48	√	-	√	-
3	Alkane	4-Methyl(trimethylene)silyloxyoctane	C_12_H_26_OSi	214.42	√	√	-	-
		1-Methyl-1-(3-phenylprop-2-enyl)oxy-1-silacyclobutane	C_13_H_18_OSi	218.36	√	√	-	-
		3,7,11,14,18-Pentaoxa-2,19-disilaeicosane	C_17_H_40_O_5_Si_2_	380.70	√	√	√	√
		1-Methyl-1-n-octyloxy-1-silacyclobutane	C_12_H_26_OSi	214.41	√	√	√	√
		3,7,11,15,18-Pentaoxa-2,19-disilaeicosane	C_17_H_40_O_5_Si_2_	380.7	√	√	-	-
		(2S,2′S)-2,2′-Bis[1,4,7,10,13-pentaoxacyclopentadecane]	C_20_H_38_O_10_	438.00	√	√	-	-
		1-(Methoxymethoxy)-3-methyl-3-hydroxybutane	C_7_H_16_O_3_	148.20	-	-	√	√
		17α,21β-28,30-Bisnorhopane	C_29_H_50_	398.71	-	-	√	√
4	Alkene	3(E)-Hydroxyimino-1,2,2,5,6,7-hexamethylbicyclo[3.2.0]hept-6-ene	C_13_H_21_ON	207.00	√	-	-	-
		9-Octadecene	C_18_H_36_	252.50	-	-	√	-
		3-Methoxyhex-1-ene	C_7_H_14_O	114.19	-	-	√	-
		H-Indene	C_9_H_8_	116.16	-	-	√	-
5	Amide	Deoxyspergualin	C_17_H_37_N_7_O_3_	387.50	√	√	-	
6	Amine	Z-Oxazolamine	C_8_H_14_N_2_O	154.21	-	-	√	√
7	Amino acids	Glycine	C_2_H_5_NO_2_	75.07	-	-	√	√
8	Aromatic oxazole compound	Benzoxazole	C_7_H_5_NO	119.12	-	-	√	√
9	Azide	Pyridine-4-carbohydrazide	C_12_H_14_Cl_3_N_6_O_2_Ti	428.50	-	-	√	√
10	Benzene	Benzene	C_6_H_6_	78.11	√	√	√	√
11	Carboxylic acid	9-Octadecenoic acid	C_18_H_34_O_2_	282.50	√	√	-	-
		5,8,11,14-Eicosatetraynoic acid	C_20_H_24_O_2_	296.40	√	√	-	-
		1,2-Benzenedicarboxylic acid	C_16_H_20_O_4_	276.33	√	√	-	-
		n-Hexadecanoic acid	C_16_H_32_O_2_	256.42	√	√	√	√
		9,12-Octadecadiynoic acid	C_18_H_28_O_2_	276.40	√	√	-	-
		Butanoic acid	C_10_H_22_O_2_Si	202.37	√	√	-	-
		Hexanoic acid	C_6_H_12_O_2_	1.08	-	-	√	√
		6-Benzoylhexanoic acid	C_13_H_16_O_3_	220.26	-	-	√	√
		cis-11-Eicosenoic acid	C_20_H_38_O_2_	310.50	-	-	√	√
		Tetradecanoic acid	C_14_H_28_O_2_	229.36	-	-	√	√
		Pentadecanoic acid	C_15_H_30_O_2_	242.40	-	-	√	√
		Heptadecanoic acid	C_17_H_34_O_2_	270.50	-	-	√	√
		Octadecanoic acid	C_18_H_36_O_2_	284.50	-	-	√	√
		4,7,7-Trimethyl-3-oxo-2-oxabicyclo[2.2.1]heptane-1-carboxylic acid	C_12_H_26_OSi	214.42	-	-	√	√
		Propanoic acid	C_3_H_6_O_2_	74.08	-	-	√	√
		Hexadecanoic acid	C_16_H_32_O_2_	256.42	-	-	√	√
		10,12-Tricosadiynoic acid	C_23_H_38_O_2_	346.50	-	-	√	√
12	Cyclic azines	4H-Pyrazole	C_3_H_4_N_2_	68.08	√	-	-	-
13	Cyclic carboxylic ester	3-Deoxy-d-mannoic lactone	C_6_H_10_O_5_	162.14	√	-	-	-
14	Ester	Methyl 2,2-dimethyl-3,6,9,12,15,18,21-heptaoxa-2-silatricosan-23-oate	C_18_H_38_O_9_Si	426.60	√	-	-	-
15	Ether	Ether	C_4_H_10_O	74.12	√	-	-	-
16	Glucosinolate	Glucobrassicin	C_16_H_19_N_2_O_9_S_2_-	447.46	-	-	-	√
17	Glycoside	Galactopyranoside	C_7_H_14_O_6_	194.18	√	√	-	-
		α-D-Glucopyranoside	C_7_H_14_O_6_	194.18	√	√	-	-
		Methyl-α-D-thiogalactoside	C_7_H_14_O_6_	194.18	-	-	√	-
		Methyl 3-O-acetyl-2,4,6-tri-O-ethyl-α-d-mannopyranoside	C_13_H_22_O_8_	306.31	-	-	√	-
		Octyl-α-D-glucopyranoside	C_14_H_28_O_6_	292.37	-	-	√	-
		α-D-Galactopyranoside	C_12_H_15_NO_8_	301.25	-	-	√	-
18	Hetero cyclic dioxin	4H-1,3-Benzodioxin	C_8_H_8_O_2_	136.15	√	√	-	-
19	Heterocyclic organo oxygen	2-Vinylfuran	C_6_H_6_O	94.11	-	-	√	√
20	Isoquinoline	Papaveroline	C_16_H_14_BrNO_4_	364.19			√	√
21	Ketone	4′-Chlorobutyrophenone	C_10_H_11_ClO	182.64	√	-	-	-
		4H-Pyran-4-one	C_5_H_4_O_2_	96.08	√	-	-	-
		6-Amino-1-α-d-ribofuranosylimidazo[4,5-c]pyridin-4(5H)-one	C_19_H_14_N_2_O_2_	302.3	√	-	-	-
		8,8-Dimethyl-3-(4-methyl-5-oxo-2,5-dihydrofuran-2-yloxymethylene)-3a,4,6,7,8,8b-hexahydro-3H-indeno[1,2-b]furan-2,5-dione	C_39_H_40_O_12_	700.73	√	-	-	-
		5,6-Epoxy-6-methyl-2-heptanone	C_8_H_14_O	142.20	√	-	-	-
		Propan-1-one	C_12_H_15_NO_2_	205.25	√	-	-	-
		2-Azetidinone	C_3_H_5_NO	71.08	√	-	-	-
		4-Carbomethoxy-4-[2-(2-carbomethoxyvinyl)benzyl]-3-methoxy-2,5-cyclohexadien-1-one	C_20_H_20_O_6_	356.37	√	-	-	-
		Dihydroxyacetone	C_3_H_6_O_3_	90.08	-	-	√	√
		2H-Pyran-2-one	C_5_H_4_O_2_	96.08	-	-	√	√
		12-Oxatricyclo [4.4.3.0(1,6)] tridecane-3,11-dione	C_12_H_16_O_3_	208.25	-	-	√	√
		Androstan-3-one	C_19_H_30_O	274.40	-	-	√	√
22	Lanostane skeleton	Cucurbitacin b	C_64_H_90_O_16_	1115.40	-	-	√	√
23	Sugars	α-D-Glucopyranose	C_6_H_12_O	180.16	√	√	√	√
		Rhamnose	C_6_H_12_O_5_	164.07	√	√	-	-
		D-Allose	C_6_H_12_O_6_	180.16	-	-	√	√
		3-O-Methyl-d-glucose	C_7_H_14_O_6_	194.18	√	√	√	√
		1,6-Anhydro-2,3-O-isopropylidene-á-D-mannopyranose	C_9_H_14_O_5_	202.20	√	√	-	-
		Melezitose	C_12_H_22_O_11_	342.30	√	√	√	√
		Maltose	C_12_H_22_O_11_	342.30	√	√	-	-
		α-D-Galactopyranose	C_26_H_42_O_11_	530.60	√	√	√	√
24	Organo sulphur compound	Diphenyl sulfone	C_12_H_10_O_2_S	218.27	√	√	√	√
25	Phosphonic acid	Phosphonic acid	H_3_PO_3_	80.98	√	√	-	-
26	Triene	4-Hexyl-1-(7-methoxycarbonylheptyl)bicyclo[4.4.0]deca-2,5,7-triene	C_25_H_40_O_2_	372.60	√	√	-	-

ML: molecular weight; HS: highly susceptible; SC: susceptible check; HR: highly resistant; RC: susceptible check.

## Data Availability

Data presented in this experiment are contained within the article. The raw data can be obtained from the corresponding authors upon request due to privacy.

## Supplementary Materials

The following supporting information can be downloaded at: https://www.mdpi.com/article/10.3390/metabo13020165/s1, Table S1: Grouping of horse gram germplasm with respect to HgYMV infestation.
