# Supplementary material for: Comparative Metabolomic Profiling of Horse Gram (Macrotyloma uniflorum (Lam.) Verdc.) Genotypes for Horse Gram Yellow Mosaic Virus Resistance
Source: Metabolites. 2023 Jan 23;13(2):165. doi: 10.3390/metabo13020165 (PMC9960754; doi:10.3390/metabo13020165)
Supplement: Supplementary file 1 [file metabolites-13-00165-s001.zip › metabolites-2143276-supplementary.pdf]

Supplementary Table S1. Grouping of horse gram germplasm with respect to HgYMV infestation

| **S.No** | Genotype | PDI | Rating | Group | **S.No** | Genotype | PDI | Rating | Group | **S.No** | Genotype | PDI | Rating | Group |
|---|---|---|---|---|---|---|---|---|---|---|---|---|---|---|
| **1** | PLS 6001 | 47.50 | 7 | S | **26** | PLS 6040 | 46.75 | 7 | S | **51** | PLS 6080 | 33.75 | 6 | S |
| **2** | PLS 6002 | **1.42** | 1 | HR | **27** | PLS 6041 | 39.50 | 7 | S | **52** | PLS 6081 | 48.50 | 7 | S |
| **3** | PLS 6003 | 46.25 | 7 | S | **28** | PLS 6043 | 17.00 | 5 | MS | **53** | PLS 6082 | 18.75 | 5 | MS |
| **4** | PLS 6004 | 19.75 | 5 | MS | **29** | PLS 6046 | 48.00 | 7 | S | **54** | PLS 6083 | 48.75 | 7 | S |
| **5** | PLS 6005 | 18.75 | 5 | MS | **30** | PLS 6047 | 12.50 | 4 | MR | **55** | PLS 6085 | 42.50 | 7 | S |
| **6** | PLS 6006 | 19.50 | 5 | MS | **31** | PLS 6048 | 18.75 | 5 | MS | **56** | PLS 6088 | 37.50 | 7 | S |
| **7** | PLS 6007 | 18.75 | 5 | MS | **32** | PLS 6049 | 29.25 | 6 | S | **57** | PLS 6089 | 18.75 | 5 | MS |
| **8** | PLS 6008 | 12.00 | 4 | MR | **33** | PLS 6050 | 48.75 | 7 | S | **58** | PLS 6090 | 17.00 | 5 | MS |
| **9** | PLS 6009 | 18.75 | 5 | MS | **34** | PLS 6051 | 17.00 | 5 | MS | **59** | PLS 6092 | 12.25 | 4 | MR |
| **10** | PLS 6013 | 12.00 | 4 | MR | **35** | PLS 6052 | 43.00 | 7 | S | **60** | PLS 6094 | 48.75 | 7 | S |
| **11** | PLS 6014 | 12.25 | 4 | MR | **36** | PLS 6059 | 37.50 | 7 | S | **61** | PLS 6095 | 66.25 | 7 | S |
| **12** | PLS 6015 | 46.25 | 7 | S | **37** | PLS 6060 | 12.50 | 4 | MR | **62** | PLS 6097 | 19.25 | 5 | MS |
| **13** | PLS 6016 | 19.00 | 5 | MS | **38** | PLS 6061 | 43.75 | 7 | S | **63** | PLS 6099 | 32.00 | 6 | S |
| **14** | PLS 6019 | 18.75 | 5 | MS | **39** | PLS 6062 | 18.75 | 5 | MS | **64** | PLS 6102 | 37.50 | 7 | S |
| **15** | PLS 6021 | 17.00 | 5 | MS | **40** | PLS 6063 | 18.75 | 5 | MS | **65** | PLS 6103 | 46.25 | 7 | S |
| **16** | PLS 6023 | 18.75 | 5 | MS | **41** | PLS 6064 | 19.25 | 5 | MS | **66** | PLS 6104 | 18.00 | 5 | MS |
| **17** | PLS 6025 | 15.75 | 5 | MS | **42** | PLS 6066 | 43.75 | 7 | S | **67** | PLS 6105 | 16.75 | 5 | MS |
| **18** | PLS 6030 | 19.00 | 5 | MS | **43** | PLS 6068 | 39.25 | 7 | S | **68** | PLS 6106 | 18.75 | 5 | MS |
| **19** | PLS 6033 | 40.00 | 7 | S | **44** | PLS 6069 | 42.50 | 7 | S | **69** | PLS 6107 | 12.50 | 4 | MR |
| **20** | PLS 6034 | 16.00 | 5 | MS | **45** | PLS 6071 | 16.00 | 5 | MS | **70** | PLS 6109 | 43.75 | 7 | S |
| **21** | PLS 6035 | 18.25 | 5 | MS | **46** | PLS 6072 | 48.75 | 7 | S | **71** | PLS 6110 | 16.00 | 5 | MS |
| **22** | PLS 6036 | 18.75 | 5 | MS | **47** | PLS 6073 | 41.00 | 7 | S | **72** | PLS 6111 | 17.00 | 5 | MS |
| **23** | PLS 6037 | 16.00 | 5 | MS | **48** | PLS 6074 | 17.00 | 5 | MS | **73** | PLS 6112 | 46.25 | 7 | S |
| **24** | PLS 6038 | 19.25 | 5 | MS | **49** | PLS 6077 | 18.75 | 5 | MS | **74** | PLS 6113 | 37.50 | 6 | S |
| **25** | PLS 6039 | 11.50 | 4 | MR | **50** | PLS 6078 | 12.50 | 4 | MR | **75** | PLS 6114 | 18.75 | 5 | MS |

Supplementary Table S1 Contd.,

| S.No | Genotype | PDI | Rating | Group | S.No | Genotype | PDI | Rating | Group | S.No | Genotype | PDI | Rating | Group |
|---|---|---|---|---|---|---|---|---|---|---|---|---|---|---|
| **76** | PLS 6115 | 18.00 | 5 | MS | **101** | PLS 6183 | 19.50 | 5 | MS | **126** | PLS 6224 | 12.50 | 4 | MR |
| **77** | PLS6116 | 12.50 | 4 | MR | **102** | PLS 6184 | 33.75 | 6 | S | **127** | PLS 6226 | 19.00 | 5 | MS |
| **78** | PLS 6118 | 37.50 | 7 | S | **103** | PLS 6185 | 17.00 | 5 | MS | **128** | PLS 6227 | 12.75 | 4 | MR |
| **79** | PLS 6119 | 46.25 | 7 | S | **104** | PLS 6186 | 18.75 | 5 | MS | **129** | PLS 6228 | 26.25 | 6 | S |
| **80** | PLS 6120 | 42.50 | 7 | S | **105** | PLS 6190 | 19.00 | 5 | MS | **130** | PLS 6229 | 28.50 | 6 | S |
| **81** | PLS 6121 | 48.75 | 7 | S | **106** | PLS 6192 | 29.75 | 6 | S | **131** | PLS 6230 | 18.00 | 5 | MS |
| **82** | PLS 6125 | 19.00 | 5 | MS | **107** | PLS 6193 | 37.50 | 6 | S | **132** | PLS 6231 | 12.50 | 4 | MR |
| **83** | PLS 6131 | 18.75 | 5 | MS | **108** | PLS 6194 | 75.00 | 9 | HS | **133** | PLS 6232 | 16.00 | 5 | MS |
| **84** | PLS 6135 | 14.25 | 4 | MR | **109** | PLS 6196 | 41.00 | 7 | S | **134** | PLS 6233 | 18.75 | 5 | MS |
| **85** | PLS 6140 | 48.75 | 7 | S | **110** | PLS 6197 | 18.50 | 5 | MS | **135** | PLS 6234 | 12.50 | 3 | MR |
| **86** | PLS 6141 | 18.75 | 5 | MS | **111** | PLS 6199 | 26.25 | 6 | S | **136** | PLS 6236 | 36.25 | 7 | S |
| **87** | PLS 6142 | 18.75 | 5 | MS | **112** | PLS 6200 | 48.75 | 7 | S | **137** | PLS 6237 | 48.75 | 7 | S |
| **88** | PLS 6150 | 16.50 | 5 | MS | **113** | PLS 6201 | 12.50 | 4 | MR | **138** | PLS 6240 | 36.25 | 7 | S |
| **89** | PLS 6151 | 19.00 | 5 | MS | **114** | PLS 6202 | 46.25 | 7 | S | **139** | PLS 6242 | 17.00 | 5 | MS |
| **90** | PLS 6154 | 16.50 | 5 | MS | **115** | PLS 6205 | 43.75 | 7 | S | **140** | PLS 6244 | 48.75 | 7 | S |
| **91** | PLS 6161 | 29.50 | 6 | S | **116** | PLS 6206 | 47.50 | 7 | S | **141** | PLS 6245 | 12.50 | 4 | MR |
| **92** | PLS 6164 | 33.75 | 7 | S | **117** | PLS 6208 | 18.00 | 5 | MS | **142** | PLS 6246 | 42.50 | 7 | S |
| **93** | PLS 6165 | 17.00 | 5 | MS | **118** | PLS 6211 | 18.75 | 5 | MS | **143** | PLS 6247 | 13.25 | 4 | MR |
| **94** | PLS 6168 | 18.75 | 5 | MS | **119** | PLS 6212 | 12.25 | 4 | MR | **144** | PLS 6250 | 18.75 | 5 | MS |
| **95** | PLS 6169 | 33.75 | 7 | S | **120** | PLS 6213 | 43.75 | 7 | S | **145** | PLS 6251 | 42.50 | 7 | S |
| **96** | PLS 6172 | 46.25 | 7 | S | **121** | PLS 6216 | 26.25 | 6 | S | **146** | PLS 6252 | 26.25 | 6 | S |
| **97** | PLS 6175 | 26.25 | 6 | S | **122** | PLS 6217 | 16.00 | 5 | MS | **147** | PLS 6253 | 19.00 | 5 | MS |
| **98** | PLS 6177 | 48.75 | 7 | S | **123** | PLS 6218 | 17.00 | 5 | MS | **148** | PLS 6255 | 48.75 | 7 | S |
| **99** | PLS 6179 | 17.00 | 5 | MS | **124** | PLS 6219 | 18.50 | 5 | MS | **149** | PLS 6256 | 43.75 | 7 | S |
| **100** | PLS 6181 | 13.25 | 4 | MR | **125** | PLS 6221 | 18.75 | 5 | MS | **150** | PLS 6258 | 46.25 | 7 | S |

Supplementary Table S1 Contd.,

| **S.No** | Genotype | PDI | Rating | Group | **S.No** | Genotype | PDI | Rating | Group | **S.No** | Genotype | PDI | Rating | Group |
|---|---|---|---|---|---|---|---|---|---|---|---|---|---|---|
| **151** | PLS 6260 | 18.00 | 5 | MS | **176** | HG 8 | 18.75 | 5 | MS | **201** | HG 59 | 46.25 | 7 | S |
| **152** | PLS 6261 | 46.25 | 7 | S | **177** | HG 9 | 17.00 | 5 | MS | **202** | HG 61 | 22.50 | 7 | S |
| **153** | PLS 6262 | 18.75 | 5 | MS | **178** | HG 9A | 42.50 | 7 | S | **203** | HG 63 | 18.00 | 5 | MS |
| **154** | PLS 6263 | 12.50 | 4 | MR | **179** | HG 12 | 46.25 | 7 | S | **204** | HG 67 | 12.50 | 4 | MR |
| **155** | PLS 6266 | 29.00 | 6 | S | **180** | HG 14 | 43.75 | 7 | S | **205** | HG 68 | 26.25 | 6 | S |
| **156** | PLS 6268 | 23.25 | 6 | S | **181** | HG 18 | 16.00 | 5 | MS | **206** | HG 72 | 42.50 | 7 | S |
| **157** | PLS 6269 | 12.50 | 4 | MR | **182** | HG 19 | 18.25 | 5 | MS | **207** | HG 78 | 44.75 | 7 | S |
| **158** | PLS 6270 | 23.75 | 6 | S | **183** | HG 21 | 48.75 | 7 | S | **208** | HG 79 | 19.00 | 5 | MS |
| **159** | PLS 6272 | 48.75 | 7 | S | **184** | HG 23 | 16.00 | 5 | MS | **209** | HG 80 | 42.50 | 7 | S |
| **160** | PLS 6275 | 18.75 | 5 | MS | **185** | HG 27 | 46.25 | 7 | S | **210** | HG 85 | 14.00 | 5 | MS |
| **161** | PLS 6278 | 12.50 | 4 | MR | **186** | HG 28 | 12.50 | 4 | MR | **211** | HG 86 | 46.25 | 7 | S |
| **162** | PLS 6279 | 42.50 | 7 | S | **187** | HG 30 | 18.00 | 5 | MS | **212** | HG 90 | 12.50 | 4 | MR |
| **163** | PLS 6280 | 27.50 | 6 | S | **188** | HG 31 | 18.75 | 5 | MS | **213** | HG 92 | 47.75 | 7 | S |
| **164** | PLS 6281 | 17.00 | 5 | MS | **189** | HG 34 | 40.00 | 7 | S | **214** | HG 93 | 18.75 | 5 | MS |
| **165** | [PLS 6282 | 18.75 | 5 | MS | **190** | HG 35 | 43.75 | 7 | S | **215** | HG 94 | 13.00 | 5 | MS |
| **166** | PLS 6055 | 43.75 | 7 | S | **191** | HG 36 | 48.75 | 7 | S | **216** | HG 95 | 17.00 | 5 | MS |
| **167** | PLS 6132 | 48.75 | 7 | S | **192** | HG 37 | 26.25 | 6 | S | **217** | HG 96 | 48.75 | 7 | S |
| **168** | PLS 6117 | 19.00 | 5 | MS | **193** | HG 38 | 16.00 | 5 | MS | **218** | HG 101 | 12.50 | 4 | MR |
| **169** | PLS 6096 | 12.50 | 4 | MR | **194** | HG 41 | 18.75 | 5 | MS | **219** | HG 112 | 42.50 | 7 | S |
| **170** | PLS 6018 | 18.75 | 5 | MS | **195** | HG 43 | 45.00 | 7 | S | **220** | HG 114 | 19.00 | 5 | MS |
| **171** | PLS 6070 | 46.25 | 7 | S | **196** | HG 47 | 48.75 | 7 | S | **221** | HG 115 | 42.75 | 7 | S |
| **172** | HG 2 | 40.00 | 7 | S | **197** | HG 50 | 18.75 | 5 | MS | **222** | HG 116 | 46.75 | 7 | S |
| **173** | HG 4 | 48.75 | 7 | S | **198** | HG 54 | 12.50 | 4 | MR | **223** | HG 119 | 44.25 | 6 | S |
| **174** | HG 5 | 18.75 | 5 | MS | **199** | HG 57 | 18.75 | 5 | MS | **224** | HG 120 | 12.50 | 4 | MR |
| **175** | HG 5A | 18.00 | 5 | MS | **200** | HG 58 | 40.00 | 6 | S | **225** | HG 121 | 43.75 | 7 | S |

Supplementary Table S1 Contd.,

| **S.No** | Genotype | PDI | Rating | Group | **S.No** | Genotype | PDI | Rating | Group | **S.No** | Genotype | PDI | Rating | Group |
|---|---|---|---|---|---|---|---|---|---|---|---|---|---|---|
| **226** | HG 121-4 | 18.75 | 5 | MS | **235** | 2416082 | 12.50 | 4 | MR | **244** | 2447523 | 12.50 | 4 | MR |
| **227** | HG 122 | 31.25 | 7 | S | **236** | 2416112 | 43.75 | 7 | S | **245** | 2447889 | 48.75 | 7 | S |
| **228** | HG 125 | 48.75 | 7 | S | **237** | 2416171 | 46.25 | 7 | S | **246** | 2448987 | 46.25 | 7 | S |
| **229** | HG 204 | 42.50 | 7 | S | **238** | 2416446 | 42.50 | 7 | S | **247** | 2448984 | 50.00 | 7 | S |
| **230** | HG 376 | 13.25 | 4 | MR | **239** | 2448985 | 43.00 | 7 | S | **248** | 2449350 | 18.75 | 5 | MS |
| **231** | HG 473 | 42.50 | 7 | S | **240** | 2449349 | 19.00 | 5 | MS | **249** | 2447858 | 19.00 | 5 | MS |
| **232** | 2415016 | 19.00 | 5 | MS | **241** | 2449351 | 48.75 | 7 | S | **250** | 2449320 | 43.75 | 7 | S |
| **233** | 2415443 | 18.75 | 5 | MS | **242** | 2449352 | 31.25 | 7 | S | **251** | PAIYUR 2 | 12.25 | 4 | MR |
| **234** | 2415806 | 17.00 | 5 | MS | **243** | 2447527 | 46.75 | 7 | S | **252** | CRIDA 1-18R | 12.50 | 3 | MR |
| | | | | | | | | | | | HG 22: SC | 75.00 | 7 | HS |

S- Susceptible MS: Moderately susceptible HS: Highly susceptible MR: Moderately resistant HR: Highly Resistant

SC: Susceptible check
